# Stepwise Incremental Hemodialysis and Low-Protein Diet Supplemented with Keto-Analogues Preserve Residual Kidney Function: A Randomized Controlled Trial †

**DOI:** 10.3390/nu17152422

**Published:** 2025-07-24

**Authors:** Piyawan Kittiskulnam, Khajohn Tiranathanagul, Paweena Susantitaphong, Jeerath Phannajit, Yuda Chongpison, Pagaporn Asavapujanamanee, Bongkod Surattichaiyakul, Kullaya Takkavatakarn, Pisut Katavetin, Kamonchanok Metta, Kearkiat Praditpornsilpa

**Affiliations:** 1 Division of Internal Medicine-Nephrology, Department of Medicine, Faculty of Medicine, Chulalongkorn University, Bangkok 10330, Thailand; piyawankitti@gmail.com; 2 Division of Nephrology, Department of Medicine, Faculty of Medicine, Chulalongkorn University, Bangkok 10330, Thailand; khajohn_t@hotmail.com (K.T.); pesancerinus@hotmail.com (P.S.); jeerathp@gmail.com (J.P.); koykullaya@hotmail.com (K.T.); pkatavetin@yahoo.com (P.K.); kamonchanok.met@gmail.com (K.M.); 3 Special Task Force for Activating Research in Renal Nutrition (Renal Nutrition Research Group), Office of Research Affairs, Chulalongkorn University, Bangkok 10330, Thailand; 4 Center of Excellence for Metabolic Bone Disease in CKD Patients, Faculty of Medicine, Chulalongkorn University, Bangkok 10330, Thailand; 5 Division of Clinical Epidemiology, Department of Medicine, Faculty of Medicine, Chulalongkorn University, Bangkok 10330, Thailand; 6 Center of Excellence in Biostatistics, Research Affairs, Faculty of Medicine, Chulalongkorn University, Bangkok 10330, Thailand; yuda.c@chula.ac.th; 7 Hemodialysis Center BENCHAKITTI–MDCU, Benjakitti Park Hospital, Bangkok 10330, Thailand; soi-korn@hotmail.com; 8 Division of Nephrology, Bhumirajanagarindra Kidney Institute, Bangkok 10330, Thailand; bong_kod@hotmail.com

**Keywords:** incremental hemodialysis, low-protein diet, keto-analogue, residual kidney function

## Abstract

**Background:** Rapid loss of residual kidney function (RKF) is associated with unfavorable outcomes. We conducted an RCT to compare the effects on RKF preservation of incremental HD between once-weekly HD (1-WHD) and twice-weekly HD (2-WHD). **Methods:** ESKD patients with an eGFR of 5–10 mL/min/1.73 m^2^ and urine output of ≥800 mL/day were randomly assigned to receive either once-weekly HD (1-WHD) or twice-weekly HD (2-WHD) for 12 months. Patients in the 1-WHD group were prescribed once-weekly HD combined with low-protein diet (0.6 g/kg/day) supplemented with keto-analogues (KAs) 0.12 g/kg/day. In the 2-WHD group, patients received twice-weekly HD with a regular-protein diet. Primary outcomes were changes in RKF by renal clearance and urine volume. Nutritional status, muscle parameters, and quality of life (QoL) were also assessed. **Results:** A total of 30 incident HD patients were randomized. Baseline RKF, urine volume, and demographic were not different between groups. After 3 months, urine volume was significantly higher in the 1-WHD group than in the 2-WHD group (1921 ± 767 mL/day vs. 1305 ± 599 mL/day, *p* = 0.02), and these significant findings persisted throughout the entire study period. For RKF, 1-WHD also had a lesser decline in urinary urea (CUrea) and creatinine clearance (CCr) than 2-WHD, with statistically significant differences observed from months 6–12. By month 6, the 1-WHD group exhibited significantly higher CUrea and CCr compared to the 2-WHD group, with CUrea at 3.2 ± 2.3 vs. 1.7 ± 1.0 mL/min (*p* = 0.03) and CCr at 5.9 ± 3.6 vs. 3.8 ± 1.4 mL/min (*p* = 0.04), respectively. Serum albumin levels, skeletal muscle mass, anemia status, metabolic parameters, protein-bound uremic toxins, and QoL scores were comparable between the two groups. **Conclusions:** Incremental HD, starting with once-weekly HD combined with protein restriction supplemented with KAs, appears to better preserve RKF among incident HD patients compared to twice-weekly HD with a regular-protein diet. This HD regimen was also associated with safety in metabolic and nutritional profiles.

## 1. Introduction

Preserving residual kidney function (RKF) in patients with end-stage kidney disease (ESKD) undergoing hemodialysis (HD) is crucial due to its significant benefits [1]. RKF contributes to better volume control, enhanced clearance of middle molecules and protein-bound uremic toxins, and improved patient survival [2]. A cohort study of 6538 patients initiating maintenance HD over four years demonstrated that the rate of RKF decline within the first year correlated incrementally with increased all-cause mortality [3]. These advantages underscore the importance of preserving RKF in chronic HD patients [4].

Several strategies have been implemented to maintain RKF, including avoiding nephrotoxic agents, minimizing excessive ultrafiltration, preventing intradialytic hypotension, and utilizing high-flux biocompatible dialyzers with ultrapure water. Despite these efforts, initiating HD with more frequent sessions often accelerates RKF decline. Studies on frequent nocturnal hemodialysis (FNH), performed five times per week, showed that urine volume declined to zero in 52% and 67% of patients at months 4 and 12, respectively, suggesting that frequent HD accelerates RKF loss [5].

Unlike the conventional thrice-weekly regimen, often prescribed from the outset, incremental HD tailors dialysis frequency to the patient’s RKF. A recent study demonstrated that reducing HD frequency from thrice to twice weekly was feasible using the higher contribution assigned to residual urea clearance (Kru) by the 2015 KDOQI guidelines [6]. By reducing the physical, time, and cardiovascular burdens of dialysis, incremental HD may improve overall patient well-being and quality of life. A longitudinal cohort study of 23,645 patients initiating HD found that those on an incremental regimen (initially twice-weekly) demonstrated better preservation of renal urea clearance and urine volume than those on conventional thrice-weekly HD, suggesting a safe approach to preserving RKF [7]. Meta-analyses in 2019 [8] and 2023 [9] further showed that twice-weekly incremental HD slowed RKF decline compared to thrice-weekly HD, reducing hospitalization rates and delaying the need for full-dose HD without increasing mortality risk.

Our group reported a case series of 11 incident hemodialysis (HD) patients with preserved residual kidney function (RKF), defined as a renal urea clearance ≥ 3 mL/min and urine output ≥ 800 mL/day, who initiated once-weekly online hemodiafiltration (OL-HDF) combined with a low-protein diet (0.6–0.8 g/kg/day) on non-dialysis days and increased protein intake (1.2 g/kg/day) on dialysis days [10]. This once-weekly protocol, even in patients with very low RKF, was sustained for a median duration of 7 months, with some patients maintaining it for up to 24 months before requiring an increase in dialysis frequency. These findings suggest that initiating HD at the lowest feasible frequency may be a viable strategy for RKF preservation, meriting further investigation in clinical trials.

Because protein restriction is primarily used to slow the progression of CKD before dialysis initiation, lower dietary protein intake may be continued after transitioning to dialysis as a strategy to reduce blood urea nitrogen levels and mitigate uremia-related complications, which are often associated with high-protein diets [11]. Moreover, the abrupt increase in recommended protein intake from 0.6 to 0.8 g/kg/day in non-dialysis CKD to 1.1–1.2 g/kg/day at the start of hemodialysis may hasten the decline of RKF, underscoring the need for a more balanced approach to protein management during this critical transition period [12,13,14]. Notably, CKD patients who receive a low-protein diet supplemented with keto-analogues (KAs) appear to adapt more efficiently to reduced protein intake compared to those without supplementation [15]. However, the optimal level of protein intake, with or without KAs, during the dialysis transition remains to be determined.

While observational studies suggest benefits of incremental HD and low-protein diets in preserving RKF, randomized trials evaluating these strategies, individually or in combination, are lacking. To our knowledge, no ongoing RCTs are assessing their combined effect. This study addresses that gap by evaluating a stepwise incremental HD approach, starting with once-weekly HD plus a low-protein diet (0.6 g/kg/day) and KA supplementation (0.12 g/kg/day) on non-dialysis days, compared to twice-weekly HD with a regular-protein diet (1.0–1.2 g/kg/day).

## 2. Materials and Methods

### 2.1. Study Design and Participants

Inclusion criteria were age ≥ 18 years, stable CKD stage 5ND with an estimated glomerular filtration rate (eGFR) of 5–10 mL/min/1.73 m^2^ based on the CKD-EPI creatinine equation, urine output ≥ 800 mL/day, willingness to participate, and ability to provide informed consent.

Exclusion criteria included rapid GFR decline (defined as an eGFR decrease >10 mL/min/1.73 m^2^ over the 6 months preceding enrollment), presence of wasting diseases such as cancer, tuberculosis, or HIV infection, active infection or inflammation indicated by C-reactive protein levels >10 mg/L, gastrointestinal conditions preventing adherence to nutritional therapy (e.g., persistent nausea, vomiting, dysphagia, chronic diarrhea, or malabsorption), prior kidney transplantation with ongoing immunosuppressive therapy, pregnancy, persistent hypercalcemia (serum calcium ≥ 10.5 mg/dL), and body mass index (BMI) ≥ 35 kg/m^2^.

The primary outcome was the change in urine urea clearance at 12 months. The sample size was calculated using a two-sided *t*-test to detect a large effect size (Cohen’s d = 0.8), with a significance level of α = 0.05 and 80% power (β = 0.20), requiring 25 participants per group (total *n* = 50). Due to COVID-19-related constraints, an unplanned interim analysis was conducted after 30 patients (60% of the planned sample) completed follow-up. As the interim results favored the 1-WHD group (once-weekly HD with low-protein diet and keto-analogues) in preserving residual kidney function, and given ethical and practical considerations, further enrollment was halted. To address the potential inflation of type I error, a Bonferroni correction was applied, adjusting the significance level to α = 0.025.

### 2.2. Run-In Period and Randomization

**Figure 1:**
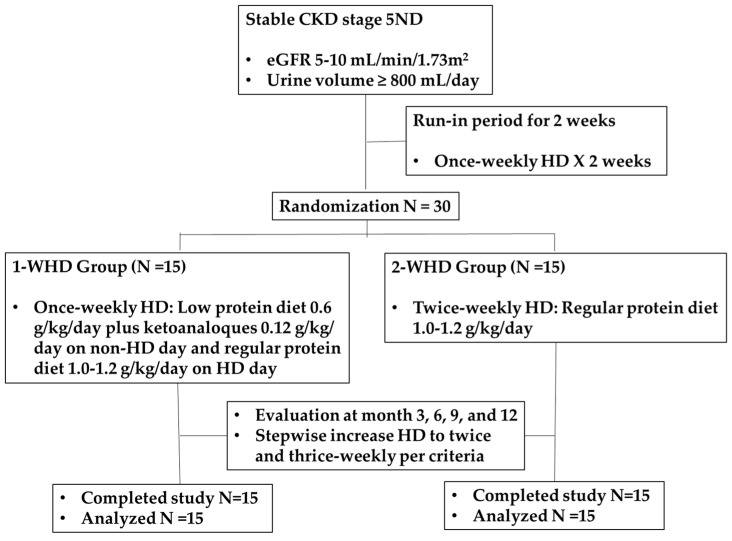
CONSORT diagram.

After screening, participants underwent a two-week run-in period, during which they received a 2 h low-flux HD session in the first week and a 4 h session in the second week (Figure 1). Throughout this period, patients were advised to maintain the same low-protein diet they had followed during the pre-dialysis phase. Patients who met the inclusion and exclusion criteria were then randomized in a 1:1 ratio into 2 groups (1-WHD and 2-WHD) using a stratified permuted block design. The 1-WHD group received once-weekly 4 h HD sessions, a low-protein diet (0.6 g/kg/day), and KAs (0.12 g/kg/day) on non-dialysis days, and a regular-protein diet (1.0–1.2 g/kg/day) on dialysis days. The 2-WHD group received twice-weekly 4 h HD sessions with a regular-protein diet. Both groups were followed for 12 months. Energy intake was targeted at 30–35 kcal/kg/day; volume management was guided by a Body Composition Monitor (Fresenius Medical Care^®®^, St. Wendel, Germany). Both groups used biocompatible high-flux dialyzers with polynephron membranes (surface area 2.1 m^2^) and ultrapure water, receiving a blood flow of 350–400 mL/min and a dialysate flow of 800 mL/min. Home blood pressure monitoring was employed to guide individualized blood pressure management, while loop diuretics were titrated to optimize urine output and support fluid balance. Regular nutritional counseling and adherence to the prescribed diet monitoring were provided by a dietitian.

### 2.3. Follow-Up Evaluation

Laboratory assessments, including blood tests and 24 h urine collections, were conducted at months 3, 6, 9, and 12. HD frequency was increased for fluid overload, symptoms of heart failure, acute coronary syndrome, persistent hyperkalemia (K^+^ > 5.5 mmol/L), hyperphosphatemia (PO_4_^2−^ > 5.5 mg/dL), serum albumin ≤ 3.5 g/dL, or inadequate HD (eqKt/V ≤ 1.2 in 1-WHD and standard Kt/V ≤ 2.1 in 2-WHD). Quality of life (QoL) was assessed using the Thai version of the SF-36, a validated health-related quality of life tool [16]. The 7-point Subjective Global Assessment (SGA) score was used to assess the nutritional status. Renal clearance were calculated using 24 h urine collected before the dialysis session. Muscle mass was assessed via multi-frequency bioimpedance analysis (InBody^®®^, Seoul, Republic of Korea). Indoxyl sulfate and p-cresol sulfate were analyzed by liquid chromatography–mass spectrometry. eqKt/V and nPCR was calculated through the urea kinetic model (UKM) [17].

### 2.4. Statistical Analysis

Descriptive statistics were presented as mean ± SD for normally distributed data and median (IQR) for non-normally distributed data. Categorical variables were reported as counts. All tests were two-sided with α = 0.05. Comparisons were made using chi-squared, *t*-tests, or Mann–Whitney U tests. SF-36 results were compared between groups at randomization and month 12, with scores transformed to a 0–100 scale. Statistical analyses were performed using SPSS Statistics, version 29 (IBM Corp., Armonk, NY, USA), and *p* < 0.05 was considered significant.

## 3. Results

### 3.1. Baseline Data of Participants

Baseline demographic and clinical characteristics, including age, sex, BMI, diabetes and hypertension prevalence, laboratory parameters, and nutritional status, were comparable between the two groups (Table 1). The mean age was 58.0 ± 19.3 years in 1-WHD and 61.9 ± 16.8 years in 2-WHD (*p* = 0.56). The mean BMI was 24.0 ± 3.8 kg/m^2^ in 1-WHD and 23.5 ± 3.6 kg/m^2^ in 2-WHD (*p* = 0.71). Baseline urine volume was 2226 ± 743 mL/day in the 1-WHD group and 1690 ± 985 mL/day in the 2-WHD group (*p* = 0.10). Renal urea and creatinine clearance were comparable, and there were no significant differences (Table 1). Serum albumin levels were 3.8 ± 0.3 g/dL in the 1-WHD group and 3.7 ± 0.3 g/dL in the 2-WHD group (*p* = 0.12). Baseline eGFR was comparable between groups (6.9 ± 1.4 vs. 6.2 ± 1.9 mL/min/1.73 m^2^; *p* = 0.20). No significant differences were observed in p-cresol, or indoxyl sulfate. However, β2-microglobulin (B2M) levels were significantly higher in the 2-WHD group (18.7 ± 4.7 mg/L vs. 14.9 ± 3.0 mg/L; *p* = 0.02). There were no significant differences in skeletal muscle mass between the two groups.

### 3.2. Residual Kidney Function (RKF) and Urine Volume

Both 1-WHD and 2-WHD experienced a decline in RKF following the initiation of HD. However, by month 6, 1-WHD exhibited significantly higher urine urea clearance (CUrea) compared to 2-WHD (3.2 ± 2.3 mL/min vs. 1.7 ± 1.0 mL/min, *p* = 0.03). This difference remained significant through month 12 (Table 2). The average rate of decline in CUrea from month 0 to month 12 in 1-WHD and 2-WHD was 0.12 mL/min/month and 0.07 mL/min/month, respectively. Similarly, from month 6 onward, 1-WHD demonstrated significantly higher urine creatinine clearance (CCr) than 2-WHD (5.9 ± 3.6 mL/min vs. 3.8 ± 1.4 mL/min, *p* = 0.04), with this trend continuing through month 12. In terms of residual urine volume, both groups showed a decline after HD initiation. Starting at month 3, 1-WHD had a significantly greater urine volume than 2-WHD (1921 ± 767 mL/day vs. 1305 ± 599 mL/day, *p* = 0.02), a significant difference that persisted through month 12. The average rate of decline in urine volume from month 0 to month 12 in 1-WHD and 2-WHD was 40.7 mL/month and 67.7 mL/month, respectively. The normalized protein catabolic rate (nPCR) was significantly lower in the 1-WHD group than in the 2-WHD group at all timepoints, except at month 9 (0.90 ± 0.24 vs. 1.05 ± 0.31; *p* = 0.15).

### 3.3. Laboratory Results, β2 Microglobulin (β_2_M) and Protein-Bound Uremic Toxin

There were no significant differences between the two groups in hemoglobin, pre-HD blood urea nitrogen (BUN), creatinine, phosphate, and uric acid, potassium, or calcium levels, despite the calcium content of KAs (Table 3). Serum potassium levels were well controlled and serum albumin levels were well maintained in both groups. No significant differences were observed in p-cresol, indoxyl sulfate, or β_2_M levels.

### 3.4. Skeletal Muscle Mass, Ultrafiltration Rate per HD Session, and Furosemide Dose

At study completion, nutritional status assessed by the 7-point Subjective Global Assessment (SGA) did not significantly differ from baseline in either group. Skeletal muscle mass was well maintained throughout the study period in both groups (Table 4), with no significant differences observed. Despite receiving hemodialysis only once weekly, the 1-WHD group consistently exhibited lower ultrafiltration rates per kilogram of dry weight compared to the 2-WHD group at all timepoints. However, these differences were not statistically significant. Interestingly, the prescribed daily furosemide dose was consistently higher in the 2-WHD group, although none of these differences reached statistical significance (Table 4).

Adverse events included one case of arteriovenous fistula (AVF) stenosis requiring angioplasty in the 2-WHD group and one case of tunneled hemodialysis catheter exit-site infection in the 1-WHD group. No intradialytic hypotensive episodes occurred in either group, and blood pressure control was acceptable based on home BP monitoring.

### 3.5. Quality of Life

Baseline quality of life (QoL) scores were similar between groups (71.3 ± 11.5 in 1-WHD vs. 69.2 ± 11.7 in 2-WHD; *p* = 0.63). Over 12 months, QoL significantly improved in the 1-WHD group (78.4 ± 8.9 at month 12; *p* = 0.01), while a non-significant increase was observed in the 2-WHD group (74.2 ± 9.9; *p* = 0.21). At month 12, QoL scores remained comparable between groups (*p* = 0.23).

### 3.6. Incremental HD

No mortality occurred during the study period. In the 1-WHD group, five patients required an escalation in dialysis frequency from once-weekly to twice-weekly during the study period, prompted by a decline in urine urea clearance (CUrea) to less than 3 mL/min/1.73 m^2^. Of these, three patients transitioned at month 3, and two additional patients at month 6. No patients in the 2-WHD group required an increase from twice-weekly to thrice-weekly hemodialysis throughout the 12-month follow-up.

## 4. Discussion

The global prevalence of CKD and ESKD continues to rise. Thrice-weekly HD remains the standard modality for renal replacement therapy (RRT) [18], with adequacy commonly assessed by Kt/V or the urea reduction ratio. However, initiating thrice-weekly HD has been linked to rapid declines in RKF, particularly due to the abrupt shift from advanced CKD to intensive dialysis, alongside liberalized protein intake. This transition accelerates urine output loss, a key predictor of mortality [7], and imposes significant time, psychological, and financial burdens on patients and their families. For patients with preserved RKF, incremental HD may offer a preferable alternative. Several studies have shown that incremental HD more effectively maintains RKF compared to initiation of thrice-weekly HD [19,20,21]. Furthermore, the integration of incremental HD with comprehensive nutritional management therapy (NMT) is essential to optimize RKF preservation, with dietary strategies tailored to both dialysis and non-dialysis days. On non-dialysis days, such dietary strategies reduce uremic toxin production and mitigate the risk of disequilibrium syndrome associated with high pre-dialysis urea levels, while helping to maintain nutritional status. In this context, a low-protein diet (LPD) supplemented with KAs has been employed to limit the generation of uremic toxins on non-dialysis days, thereby reducing the risk of disequilibrium syndrome associated with high pre-dialysis urea levels and large urea reduction ratios, while maintaining patients’ nutritional status [22]. Supporting this approach, a recent observational study demonstrated that ESKD patients managed with incremental HD combined with an LPD and low-salt diet exhibited significantly greater preservation of RKF compared to those receiving standard thrice-weekly HD [23].

Previous studies on incremental HD have been limited to observational designs [24,25,26]. Our previous report on 11 incident hemodialysis (HD) patients [10] demonstrated that once-weekly HD can be feasible, even in patients with reduced RKF, as indicated by renal urea clearance around 3–4 mL/min. This study is the first prospective randomized controlled trial to test the hypothesis that combining incremental HD with a low-protein diet (LPD) and KA supplementation can better preserve RKF and maintain nutritional status in ESRD patients with an eGFR of 5–10 mL/min/1.73 m^2^. The study protocol allows less protein restriction (0.6 g/kg/day) for six days per week to support treatment adherence (Supplementary File S1). The unrestricted dietary protein intake on the dialysis day could improve psychological status from the limited diet choices, which may bring dietary fatigue. Furthermore, the regular-protein diet on the dialysis day can compensate for the increase in nitrogen demand from amino acid loss through the dialysis membrane and toward hypercatabolism during the dialysis session [27].

Our findings suggest that a regimen of once-weekly HD combined with a low-protein diet supplemented with KAs on non-dialysis days more effectively preserves RKF compared to a twice-weekly HD schedule with a regular-protein diet. This benefit was reflected in the better maintenance of urine output, urine urea clearance (CUrea), and urine creatinine clearance (CCr) over the study period. At baseline, although eGFR, CUrea, and CCr was comparable between groups, the 1-WHD group had a higher mean urine volume than the 2-WHD group (2226 ± 731 vs. 1690 ± 986 mL/day), though the difference was not statistically significant (*p* = 0.13) and despite having higher baseline urine output, the decline in urine volume at month 3 in the 1-WHD group was statistically lesser than the 2-WHD group. And along the course through month 12, although both groups exhibited a gradual decline in urine volume, the reduction was less pronounced in the once-weekly HD group, supporting the potential advantage of combining incremental dialysis with dietary interventions. These results are consistent with previous studies demonstrating that low-protein diets supplemented with KAs can slow the progression of renal function decline in patients with CKD [27]. Our findings reinforce the concept that a strategic integration of dietary management and individualized dialysis schedules may enhance the preservation of RKF, which is associated with improved quality of life in patients receiving maintenance HD.

Our study demonstrated that once-weekly HD combined with a low-protein diet (0.6 g/kg/day) supplemented with KAs on non-dialysis days, and a regular-protein diet on dialysis days, successfully maintained patients’ nutritional status comparable to that observed in twice-weekly HD with a regular-protein diet. Importantly, once-weekly HD was associated with better preservation of RKF over the 12-month study period. Notably, two-thirds of patients randomized to 1-WHD were able to maintain once-weekly dialysis throughout the study period of 12 months, with only five patients requiring an increase in dialysis frequency to twice weekly. Beyond clinical outcomes, once-weekly HD reduces the overall dialysis burden, improving patients’ quality of life by minimizing time commitment, travel, and physical strain associated with more frequent sessions. As CKD patients are already accustomed to a low-protein diet to delay CKD progression, this approach is more acceptable and likely to enhance adherence. From a healthcare systems perspective, particularly in low- and middle-income countries, implementing once-weekly HD in patients with preserved RKF could substantially reduce medical expenditures, alleviating financial pressures on both government healthcare budgets and individual patients. Thus, once-weekly HD with NMT offers a promising, patient-centered, and cost-effective strategy for preserving kidney function while maintaining clinical outcomes.

A key strength of our study was the use of a standardized protocol for dry weight assessment, employing a Body Composition Monitor (BCM). Accurate determination of dry weight is critical, as underestimation may lead to excessive ultrafiltration and accelerated loss of RKF. Additionally, participants received ongoing dietary counseling from experienced dietitians to support adherence to the prescribed protein intake. Nutritional status and skeletal muscle mass were closely monitored using multi-frequency bioimpedance analysis, while quality of life was systematically evaluated, enhancing the rigor of our clinical assessments. Importantly, comprehensive nutritional monitoring demonstrated that the once-weekly HD with low-protein diet supplemented with KAs effectively maintained nutritional status, including muscle mass, throughout the study.

Our study has several limitations. It was conducted at a single center with a relatively small sample size and was halted early after an unplanned interim analysis of 30 patients, prompted by observed efficacy in the 1-WHD group and logistical challenges during the COVID-19 pandemic. This early termination reduced statistical power and may have increased the risk of type I error, necessitating a conservative Bonferroni correction (α = 0.025). Although RKF appeared better preserved in the 1-WHD group, no significant differences were observed in the clearance or serum levels of middle-molecule and protein-bound uremic toxins, such as β2-microglobulin, indoxyl sulfate, and p-cresol sulfate. An additional limitation is the baseline imbalance in β2-microglobulin levels, which were significantly higher in the 2-WHD group. Although follow-up levels did not differ, this discrepancy may confound the interpretation of middle-molecule clearance and should be considered when evaluating the findings. Larger studies are needed to determine whether better RKF preservation leads to improved middle-molecule solute removal.

Furthermore, because incremental hemodialysis and the low-protein diet with KAs supplementation were applied together, their individual effects could not be distinguished. Future studies using separate intervention arms or factorial designs are needed to clarify their independent contributions. While our findings suggest that a tailored dialysis approach supported by nutritional management may help preserve RKF in selected patients, they should be interpreted in light of the study’s limitations. Larger, multi-center randomized trials are needed to confirm these findings and guide clinical implementation.

## 5. Conclusions

This is the first prospective randomized controlled trial evaluating the effect of a low-protein diet plus KAs on residual kidney function preservation in incremental HD. Our results indicate that stepwise incremental HD initiation, starting with one session per week combined with a low-protein diet supplemented with KAs, helps preserve residual kidney function and maintain nutritional status in incident HD patients. Further research with larger sample sizes and longer follow-up periods is warranted to confirm these findings and explore the underlying mechanisms.

## Figures and Tables

**Table 1:** Patient characteristics at baseline.

	1-WHD: Once-Weekly HD with Low-Protein Diet 0.6 g/kg/d Plus KAs 0.12 g/kg/day (*n* = 15)	2-WHD: Twice-Weekly HD with Regular-Protein Diet 1.0–1.2 g/kg/day (*n* = 15)	*p*
Male/Female (cases)	9/6	7/8	0.43
Age (years)	58.0 ± 19.3	61.9 ± 16.8	0.56
DM (cases)	7/15	7/15	0.99
Hypertension (cases)	12/15	10/15	0.29
Body Weight (kg)	68.0 ± 11.7	61.6 ± 10.5	0.12
BMI (kg/m^2^)	24.0 ± 3.8	23.5 ± 3.6	0.71
Serum BUN (mg/dL)	75.1 ± 19.2	78.1 ± 17.6	0.65
Serum Cr (mg/dL)	8.2 ± 2.2	8.7 ± 2.3	0.51
eGFR (mL/min/1.73 m^2^)	6.9 ± 1.4	6.2 ± 1.9	0.26
Urine Volume (mL/day)	2226 ± 743	1690 ± 985	0.10
Urine CUrea (mL/min)	4.6 ± 2.5	4.1 ± 2.8	0.55
Urine CCr (mL/min)	8.5 ± 4.2	6.9 ± 2.8	0.23
Serum Ca^2+^ (mg/dL)	8.4 ± 0.7	8.4 ± 0.7	0.90
Serum PO_4_^2−^ (mg/dL)	4.2 ± 0.7	4.6 ± 1.1	0.37
Serum K^+^ (mmol/L)	4.3 ± 0.6	4.2 ± 0.9	0.50
Serum Albumin (g/dL)	3.8 ± 0.3	3.7 ± 0.3	0.20
Serum Uric Acid (g/dL)	7.8 ± 2.3	8.0 ± 2.4	0.85
Hb (g/dL)	9.0 ± 1.1	8.7 ± 1.1	0.47
Serum HCO_3_^−^ (mmol/L)	22.3 ± 3.6	23.3 ± 3.2	0.44
iPTH (pg/mL)	376 ± 113	307 ± 172	0.21
Serum β2-microglobulin (mg/L)	14.9 ± 3.0	18.7 ± 4.7	0.02
Serum p-cresol (μg/mL)	7.6 ± 5.2	7.9 ± 5.3	0.88
Serum Indoxyl sulfate (μg/mL)	30.1 ± 13.6	28.8 ± 17.6	0.82
Skeletal Muscle Mass (kg/m^2^)	8.7 ± 3.2	9.9 ± 2.3	0.26
SGA Score: (cases)			0.15
Class A	14	11	
Class B	1	4	

**Table 2:** Residual kidney function (RKF) and urine volume.

	Month 3			Month 6			Month 9			Month 12		
	1-WHD Group	2-WHD Group	*p*	1-WHD: Group	2-WHD Group	*p*	1-WHD: Group	2-WHD Group	*p*	1-WHD: Group	2-WHD Group	*p*
Urine CUrea (mL/min)	3.8 ± 2.9	2.2 ± 1.0	0.06	3.2 ± 2.3	1.7 ± 1.0	0.03	2.9 ± 2.3	1.5 ± 0.6	0.03	3.3 ± 2.3	1.5 ± 1.0	0.01
Urine CCr (mL/min)	7.6 ± 6.4	6.1 ± 5.1	0.48	5.9 ± 3.6	3.8 ± 1.4	0.04	4.4 ± 1.8	3.1 ± 1.1	0.03	4.6 ± 1.9	2.5 ± 1.2	0.01
Urine volume (mL/day)	1921 ± 767	1305 ± 599	0.02	1859 ± 775	918 ± 468	<0.01	1645 ± 652	926 ± 411	<0.01	1738 ± 744	878 ± 464	<0.01
nPCR (g/kg/day)	0.89 ± 0.22	1.08 ± 0.27	0.04	0.83 ± 0.22	1.09 ± 0.25	0.01	0.90 ± 0.24	1.05 ± 0.31	0.15	0.86 ± 0.20	1.10 ± 0.23	0.01

**Table 3:** Laboratory results, β2 microglobulin and protein-bound uremic toxin.

	Month 3		Month 6		Month 9		Month 12	
	1-WHD Group	2-WHD Group	1-WHD: Group	2-WHD Group	1-WHD: Group	2-WHD Group	1-WHD: Group	2-WHD Group
Hb (g/dL)	9.6 ± 1.8	10.4 ± 1.1	10.7 ± 1.1	11.1 ± 1.5	10.8 ± 1.4	10.3 ± 1.0	10.9 ± 1.2	10.8 ± 0.9
BUN (mg/dL)	81.1 ± 21.3	77.7 ± 19.9	73.3 ± 14.9	73.1 ± 20.6	74.5 ± 18.8	75.8 ± 15.2	71.7 ± 18.1	68.9 ± 21.3
Cr (mg/dL)	9.9 ± 3.8	9.4 ± 2.8	9.8 ± 3.5	9.2 ± 2.5	10.3 ± 4.0	9.5 ± 2.4	10.1 ± 3.9	10.0 ± 3.0
K^+^ (mmol/L)	4.4 ± 0.5	4.2 ± 0.5	4.3 ± 0.5	4.3 ± 0.5	4.2 ± 0.5	4.2 ± 0.4	4.3 ± 0.6	4.4 ± 0.7
HCO_3_^−^ (mmol/L)	22.9 ± 3.6	22.6 ± 3.9	24.1 ± 3.9	24.5 ± 2.8	23.3 ± 2.7	22.5 ± 2.0	23.7 ± 2.5	23.7 ± 2.8
Alb (g/dL)	4.0 ± 0.3	3.9 ± 0.3	4.0 ± 0.3	3.9 ± 0.3	3.9 ± 0.3	3.7 ± 0.3	4.0 ± 0.2	3.9 ± 0.3
Ca^2+^ (mg/dL)	8.9 ± 0.8	8.8 ± 0.4	9.1 ± 1.0	9.1 ± 0.7	9.0 ± 0.8	8.7 ± 0.5	9.0 ± 1.0	8.6 ± 0.9
PO_4_^2−^ (mg/dL)	4.3 ± 1.2	5.3 ± 1.2	4.3 ± 0.8	4.3 ± 0.9	4.4 ± 1.4	4.5 ± 1.4	4.4 ± 1.8	4.1 ± 1.4
Uric acid (mg/dL)	7.9 ± 1.8	7.3 ± 1.9	7.1 ± 2.1	7.5 ± 2.0	7.1 ± 1.8	7.0 ± 1.8	6.4 ± 1.8	6.9 ± 1.2
β_2_M (mg/L)	-	-	22.9 ± 9.4	22.2 ± 5.3	-	-	23.2 ± 6.9	22.7 ± 3.8
p-cresol (μg/mL)	-	-	5.7 ± 4.8	9.6 ± 5.9	-	-	10.4 ± 9.5	9.4 ± 8.1
Indoxyl sulfate (μg/mL)	-	-	39.4 ± 18.3	38.5 ± 5.1	-	-	38.9 ± 20.9	41.5 ± 19.0

**Table 4:** Skeletal muscle mass, ultrafiltration rate per HD session, and furosemide dose.

	Month 3		Month 6		Month 9		Month 12	
	1-WHD Group	2-WHD Group	1-WHD: Group	2-WHD Group	1-WHD: Group	2-WHD Group	1-WHD: Group	2-WHD Group
Skeletal muscle mass (kg/m^2^)	8.6 ± 2.3	9.3 ± 1.7	8.6 ± 2.2	9.6 ± 2.7	8.7 ± 2.1	9.5 ± 1.8	8.7 ± 2.1	9.5 ± 1.7
UF rate per HD (mL/kg dry weight)	24.7 ± 20.2	30.8 ± 11.5	25.6 ± 18.9	33.2 ± 13.6	26.2± 15.4	36.0 ± 17.5	28.9 ± 15.3	36.4 ± 14.6
Furosemide dose (mg/day)	219 ± 305	329 ± 300	335 ± 339	370 ± 339	385 ± 318	610 ± 371	480 ± 397	729 ± 325
SGA score: (cases)								
Class A	13	13	14	13	14	13	14	14
Class B	2	2	1	2	1	2	1	1

## Data Availability

The data presented in this study are openly available in FigShare at https://doi.org/10.6084/m9.figshare.29553671.

## Supplementary Materials

The following supporting information can be downloaded at: https://www.mdpi.com/article/10.3390/nu17152422/s1, File S1: Study protocol. Refs. [18,28,29,30] are from Supplementary Materials.

## Abbreviations

The following abbreviations are used in this manuscript:

RCT	Randomized controlled trial
RKF	Residual kidney function
1-WHD	Once-weekly HD with low-protein diet (0.6 g/kg/day), and KAs (0.12 g/kg/day) on non-dialysis days, and a regular-protein diet (1.0–1.2 g/kg/day) on dialysis days
2-WHD	Twice-weekly HD with a regular-protein diet (1.0–1.2 g/kg/day)
KA	Keto-analogues
QoL	Quality of life
β_2_M	β2-microglobulin
