# Supplementary material for: Stepwise Incremental Hemodialysis and Low-Protein Diet Supplemented with Keto-Analogues Preserve Residual Kidney Function: A Randomized Controlled Trial
Source: Nutrients. 2025 Jul 24;17(15):2422. doi: 10.3390/nu17152422 (PMC12348161; doi:10.3390/nu17152422)
Supplement: Supplementary file 1 [file nutrients-17-02422-s001.zip › nutrients-3771535-supplementary.pdf]

CLINICAL STUDY PROTOCOL

Title: The Effects of Low Protein Diet Supplemented with Ketoanalogues on Preservation of Residual Kidney Function among Patients Undergoing Incremental Dialysis

Key Words: Residual kidney function, Incremental Hemodialysis, Low Protein Diet, Ketoanalogues

Rationale: Residual kidney function may confer a variety of advantages to patients receiving maintenance dialysis including clearance of middle molecule uremic toxins and better volume control. To preserve residual kidney function after initiation of hemodialysis, several strategies should be considered such as minimizing nephrotoxic agents, avoiding of intradialytic hypotension, use of high-flux biocompatible dialyzer, ultrapure water, and online hemodiafiltration (HDF).[1] The recent innovative approaches is a continuation of a low protein diet on non-dialysis day and regular to high protein diet (1-1.2 g/kg/day) on infrequent hemodialysis (HD) days.

In the United States, more than 90% of incident hemodialysis patients initiate a standardized 3-time per week dialysis prescription without intense management to preserve residual kidney function, although there are no convincing evidences that HD prescription based solely required Kt/Vurea provide benefit on patient mortality and quality of life. An alternative approach is “incremental dialysis” aims to provide personalized required HD dose with an intense management to preserve residual kidney function.[2, 3] Meta-analysis revealed that incremental dialysis (once to twice a week) allowed a slower rate of residual kidney function decline (-0.58 ml/min/month, p=0.007) compared to a full-dose dialysis and deferred the initiation of thrice weekly HD for approximately 12.1 (95% confidence interval 9.8-14.3) months.[4]

Recent study have suggested a combination of moderately low protein diet (0.6-0.8 g/kg/day) with essential amino acids or ketoanalogues upon this dialysis transition phase. Ketoanalogues reduce burden of uremic toxins given that they lack the amino group bound to the alpha carbon of their respective amino acids[5] and may alleviate the rate of nitrogen waste products removal by the remaining kidney function on non-dialysis interval. In addition, study demonstrated that a diet containing 0.6-0.8 g of protein/kg/day was safe and, when combined with ketoanalogues, was significantly associated with preservation of residual kidney function among peritoneal dialysis patients new to dialysis.[6]

Therefore, we hypothesized that combination of incremental HD and ketoanologues will better preserve residual kidney function and maintenance of nutritional status among the incident ESRD patients during the early initiation of chronic dialysis. This hypothesis has been a new concept of chronic hemodialysis initiation for ESRD patients

Purpose: We plan to conduct the first randomized Phase IIA clinical trial to investigate the efficacy of incremental hemodialysis and moderately low protein diet combined with ketoanologues

supplementation on preservation of residual kidney function, inflammatory status and nutritional parameters among incident chronic dialysis patients

Organization: Division of Nephrology, Faculty of Medicine, Chulalongkorn University, King Chulalongkorn Memorial Hospital

Objective:

Primary objective: To demonstrate superiority effect of incremental hemodialysis (HD) protocol (initiate with once-weekly HD) plus ketoanalogues-supplemented low-protein diet during non-dialysis days over the standard HD protocol (initiate with twice weekly HD) on preservation of residual renal function (renal urea clearance) at 12 months.

Secondary objectives

• To assess whether residual renal function (renal urea clearance) and residual urine volume differ between the two groups over 12 months.

• To determine and compare residual renal function (i.e., renal urea clearance) between the two groups over 12 months.

• To compare nutritional status (serum albumin, muscle mass) between the two treatment groups over 12 months.

Methods:

Sample Size Calculation:

As this is a pilot study, the sample size calculation will be based on recent suggestions for sample size estimation in pilot randomized trials by Whitehead et al[7]. With 80% power and the alpha level = 5%, 20 patients per treatment arm are required based on the non-central t distribution (NCT) approach assuming a small standardized effect size ($0.1 \le \delta < 0.3$). With an anticipated the drop-out rate of 20%, 25 patients per treatment arm are adequate. Therefore, we aim to include a total of 50 patients in the study.

Target number of Patients: 50 incident hemodialysis patients will be randomized 1: 1 according to the frequency of dialysis per week, amount of protein prescribed, and receiving of ketoanologues. Additionally, the different techniques of hemodialysis will be stratified by dialysis facilities as the followings (Fig 1):

- **Group 1:** Twice weekly HD program plus regular protein diet (1.0 g/kg/day) every day
- **Group 2:** Incremental HD program, starting from once weekly HD/HDF plus low protein diet (0.6 g/kg/day) and ketoanologues supplementation on

  non-dialysis days and regular protein diet (1.0-1.2 g/kg/day) on dialysis day

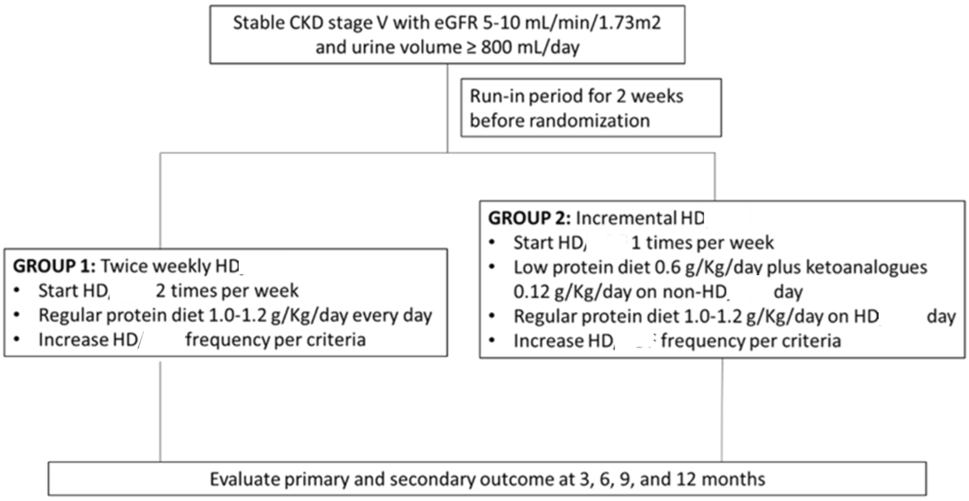

(Figure S1)

Both groups receive:

- Total energy of 30-35 kcal/Kg/day according to age group
- Use BCM method for volume management to avoid intradialytic hypotension and volume overload
- Home BP monitoring for BP control
- Instruction to collect timed urine volume and 24 hours dietary record
- Use of RAAS blockage if appropriate
- Use of loop diuretic if appropriate
- Use of biocompatible high flux dialyzer
- Use of ultrapure water dialysis
- Avoid of nephrotoxic agents and contrast media
- Herbs and other dietary supplement are not allowed
- Nutritional counselling and assessment will be regularly provided by dietitian for patients' education and diet compliance monitoring

Dialysis protocol during run-in phase before randomization:

- Low-flux HD for 2 hours * 1week, (blood flow rate 200 ml/min, dialysate flow rate 400 ml/min) then low-flux HD for 4 hours * 1week then randomization
- Regular protein diet (1.0 – 1.2 g/kg/day) every day

Inclusion Criteria:

- Age ≥ 18 years at screening

- Stable CKD stage 5 not yet on dialysis with residual kidney function 5-10 ml/min/1.73m2 by CKD-EPI equation
- Urine output ≥ 800 ml/day
- Willing to participate in the study and can provide inform consent

Exclusion Criteria:

- Rapid glomerular filtration rate (GFR) progression defined as a decline of eGFR >5 ml/min/1.73m2 by CKD-EPI equation in the prior 6 months before enrollment
- Presence of wasting diseases, cancer cachexia, tuberculosis, AIDS wasting syndrome
- Other active infection/inflammation determined by CRP >10 mg/L
- Severe gastrointestinal problem: persistent nausea/vomiting, dysphagia, chronic diarrhea, severe malabsorption
- Conditions at baseline requiring withdrawal from the study including severe protein energy wasting by SGA and MIS score, prior kidney transplantation with immunosuppressive agents or other serious medical conditions.
- Pregnancy
- Uncontrolled hypercalcemia (persistent serum Ca ≥10.5 mg/dl)
- BMI ≥ 35 kg/m2
- Hypersensitivity to the active substances or to any of the excipients of Ketosteril
- Disturbed amino acid metabolism

Criteria for remaining on once-weekly HD for Group 2 (All patients must meet all criteria before randomization) [8]

- Residual kidney function ≥3 ml/min/1.73m2 calculated by Chula urea kinetic model (UKM) formula
- Good dialysis adequacy defined by eqKt/V ≥ 1.2 /session and duration ≥4 hr/session
- Kru by Chula UKM formula <3 mL/mim
- No signs of water retention (water retention defined as required ultrafiltration rate >15 ml/kg/hour for 3 consecutive visits)
- No history of active congestive heart failure, ischemic heart disease, acute coronary syndrome, and other pulmonary symptoms
- BMI<35 kg/m2
- No persistent hyperkalemia (serum K >5.5 mEq/L) and hyperphosphatemia (serum phosphate >5.5 mg/dL) for more than 2 months
- No malnutrition (serum albumin <3.5 g/dL) and adhere to nutritional therapy
- Hemoglobin > 9 g/dL and appropriate response to anemia therapy
- Infrequent hospitalization and easily manageable other comorbid conditions

Criteria for remaining on twice-weekly HD for Group 1 and Group 2 (patients should meet first criteria plus ≥5 of other criteria) [9,10]

- Good dialysis adequacy: delivered standard Kt/V ≥ 2.1/week10
- No signs of water retention (defined as ultrafiltration rate >15 ml/kg/hour for 3 consecutive visits)
- No history of active congestive heart failure, ischemic heart disease, acute coronary syndrome, and other pulmonary symptoms

- Suitable larger body size relative to RKF
- No persistent hyperkalemia (serum K >5.5 mEq/L) and hyperphosphatemia (serum phosphate >5.5 mg/dL) for > 1 months or 2 consecutive visits (2-week interval)
- No malnutrition (serum albumin <3.5 g/dL) and adhere to nutritional therapy
- Hemoglobin >8 g/dL and appropriate response to anemia therapy (erythropoietin dose adjustment is allowed for 1 visit)
- Infrequent hospitalization and easily manageable other comorbid conditions

After the protocol approval by IRB of Faculty of Medicine, Chulalongkorn University, the participants that willing to participate the study will sign in the informed consent. They will get into run-in period and then randomization.

After randomization, the attending nephrologists at each time point (3, 6, 9, and 12 months after randomization) will determine HD frequency by criteria. The HD frequency will be increase from once per week to twice per week in Group 1, if patients fail criteria for remaining on once per week HD. The HD frequency will be increase from twice per week to thrice per week in Group 2, if patients fail criteria for remaining on twice per week HD. Patients in Group 1 receiving twice per week HD will receive thrice per week HD if patients fail criteria for remaining on twice per week HD.

Study assessments/measurements

Clinical assessments

- Definition of death: death from all causes and/or death from renal failure
- Definition of hospitalization: All cause of hospitalization

Laboratory assessments: Blood samples were drawn after overnight fasting to determine blood parameters. Serum albumin was measured in duplicate using a nephelometer and the values averaged. Muscle mass was measured by multi-frequency BIS (InBody®).

QoL assessments: The subjects will be assessed Qol by SF-36 questionnaire Thai version.

Adverse events, serious adverse events,

Adverse events leading to premature discontinuation and all events resulting in death will be assessed throughout the study.

Investigation period: 12 months 2 weeks (run-in: 2 weeks + evaluation 12 months)

Data collection: 5 points (Baseline at randomization, 3, 6, 9, and 12 months after randomization)

Measurement:

☐ **Primary outcomes** ■ **Secondary outcomes**

	Baseline	3 months	6 months	9 months	12 months

**Urinary urea clearance by timed-urine volume (24 hours preceding dialysis treatment)**	**X**	**X**	**X**	**X**	**X**
**Average of urinary urea and creatinine clearance (24 hours preceding dialysis treatment)**	**X**	**X**	**X**	**X**	**X**
**Nutritional parameters: weight, BMI, albumin, calcium, phosphate level, normalize protein catabolic rate (nPCR), SGA**	**X**	**X**	**X**	**X**	**X**
**Bioimpedance analysis**	**X**	**X**	**X**	**X**	**X**
**Other functional tests QoL (SF 36), B2microglobulin, P-cresol, indoxyl sulfate, anemia**	**X**	------	**X**	-----	**X**

Data Management Section

All data obtained in the clinical trial described in this protocol will be recorded on CRFs or trial specific entry forms. Data will be clearly documented in the Trial Documentation file which data will be collected as source data only. Laboratory data will be recorded electronically.

Confidentiality of trial documents and patient records

The study will assure that subjects' anonymity will be maintained and that their identities are protected from unauthorized parties. On CRFs or other documents, subjects would not be identified by their names, but by an identification code. The investigator would keep a subject enrolment log showing codes, names and addresses. The investigator will maintain documents e.g., subjects' written consent forms, in strict confidence. Management of clinical data will be performed in accordance with applicable standards and data cleaning procedures to ensure the integrity of the data, e.g., removing errors and inconsistencies in the data.

Safety Reporting

Adverse event reports are the critical building block to the development of the safety profile of the study. Subjects will be asked non-leading questions in general terms to determine the occurrence of AEs, according to the schedule outlined. In addition, all AEs reports spontaneously during the course of the study will be recorded. The investigator must immediately (within 24 hours of awareness) reports all SAEs, regardless of whether the investigator believes they are related to the study.

Definition of an Adverse Event: An AE can be any unfavorable and unintended sign (eg, an abnormal and clinically significant laboratory finding), symptom or disease temporally associated with the treatments without any judgment about causality. This includes any occurrence that is new in onset or aggravated in severity or frequency from the baseline condition, or abnormal results of diagnostic procedures, including laboratory test abnormalities.

An AE includes medical conditions , signs and symptoms not previously observed in the subject that emerge during the protocol-specified AE reporting period, including signs or symptoms associated with an underlying that were not present prior to the AE reporting period.

Definition of a Serious Adverse Event: All SAEs must be reported immediately. To report an SAE, the investigator must fax or email an SAE report within 24 hours of becoming aware of the serious event. Follow-up reports must be submitted in timely manner as additional information becomes available. For each SAE observed, the investigator will obtain all of the information available about the event, including (but not limited to): hospital discharge diagnosis, hospital discharge note, death certificate, appropriate laboratory finding (including autopsies and biopsy results), and clinical examinations (including radiological examinations and clinical consultation).

An SAE is any AE or suspected adverse reaction that results in any of the following outcomes:

- Death
- A life-threatening AE (ie, if in the view of the investigator, the subject was at immediate risk of death at the time of the event).
- Inpatient hospitalization or prolongation of existing hospitalization
- A persistent or significant incapacity or substantial disruption of the ability to conduct normal life functions
- A congenital anomaly or birth defect
- An important medical event (based on appropriate medical judgment, the event jeopardizes the subject and may require medical or surgical intervention to prevent one of the above-listed outcomes)

Reporting Serious Adverse Events to the Institutional Review Board/Independent Ethics Committee: The investigator is responsible of notifying Institutional Review Board (IRB), Faculty of Medicine, Chulalongkorn University of SAEs in accordance with the regulations.

Analysis plan:

For continuous variables, the descriptive statistics will be presented by number[N], mean and standard deviation [mean ± SD] for normally distributed data, and minimum, median, interquartile ranges [Q1, Q3], and maximum for non-normally distributed data. For categorical variables, the number [N] and percentage [%] of each category will be calculated for non-missing data. Missing values will also be counted and reported in each variable for both continuous and categorical variables, if applicable. All analyses will be done per treatment and per time-point."

Treatment estimates and 95% confidence intervals will be calculated and used to assess primary and secondary efficacy endpoints between randomized treatment groups.

Continuous endpoints will be analyzed using ANCOVA or non-parametric equivalents. Longitudinal analyses for continuous endpoints will be conducted using general linear mixed model (GLM). Post-hoc pairwise comparisons will be conducted using Tukey's HSD. Binary endpoints will be analyzed using chi-square tests or logistic regression. The statistical test will be two-sided with significance level $\alpha = 5\%$. Due to the exploratory nature of the pilot study, P values will be interpreted as a merely descriptive-exploratory purpose."

For all endpoints and analysis populations, the primary treatment comparisons will be simple, unadjusted, two group comparisons. If there are important imbalances in baseline characteristics, then adjusted analyses will also be performed and presented in addition to unadjusted analyses.

Data on demographics, protocol and treatment compliance, concomitant medication and safety of all included subjects (including dropouts) will be included in the trial report. Demographics, safety data at randomization and concomitant medication will be listed. Descriptive statistics will be calculated for the subject characteristics. There will be no formal comparisons of randomized treatments for demographic and baseline characteristics (i.e. no p-values), as any imbalance will by definition have occurred by chance.

Primary study endpoint analysis

The primary endpoint is the reduction of renal urea clearance after 12 months of treatment compared between the two treatment groups.

The ITT population will be the primary analysis dataset, although an analysis on the PP population will also be performed.

Secondary endpoint analysis

The following secondary efficacy endpoints will be analyzed using both the ITT and PP datasets.

Change in renal urea clearance and urine volume from randomization to each study visit will summarized by randomized treatment arms. Serious adverse events will be summarized and listed by randomized treatment arm. Descriptive statistics will be calculated for each arm. All safety endpoints will be measured at each study visit, and use a per protocol population. Serum albumin, muscle mass, nutritional status, will be summarized and compared between treatment arms. SF-36 results will be summarized in each domain and in physical and mental component scores by treatment arm, and compared by randomized treatment arm at randomization. Raw scores from questions in each quality of life domain will be transformed to a 0 to 100 scale according to the following formula: Transformed score = [(actual raw score – lowest possible raw score)/ (highest possible raw score – lowest possible raw score)] x 100].

The intention to treat (ITT) population

The ITT population is defined as all participants who are randomized. Participants will be compared as randomized regardless of the treatment received. The following describes a framework for the analysis of the primary endpoint based on a particular handling of anticipated events:

In the event the patient dies the patient will be considered to have missing urea clearance and urine volume after that time point. In the event the patient become lost to follow up the latest available parameters will be used for the later time points. If the data at any time points are missing, the latest available parameters before that time points will be used instead.

Per protocol (PP) population

The PP population is defined as all participants included in the ITT population excluding those who deviate from the randomly assigned protocol for any reason. Analyses will be based on available data

and according to the treatment received, and patients will be censored once they cease their randomized treatment.

Addendum:

Ketosteril 1 tablet is composed of

-A-ketoanalogues of BCAA (of isoleucine 67 mg, leucine 101 mg, valine 86 mg)

- A-ketoanalogues of EAA (phenylalanine 68 mg, methionine 59 mg)

-EAA (L-histidine 38 mg, L-threonine 53 mg, L-lysine 105mg, L-tryptophan23 mg)

-Non-EAA (L-tyrosine 30 mg)

-Total nitrogen 36 mg (equivalent to protein 225 mg)

-Total calcium content 50 mg, total KA/EAA 630 mg

Chula UKM formula:

$$C = C_0 \cdot e^{-k \cdot t} = C_0 \cdot e^{\frac{K_d \cdot t}{V}}$$

$$\frac{K_d \cdot t}{V} = ln\left(\frac{C_0}{C}\right)$$

- In is the natural logarithm;
- C0 is urea concentration at the start of dialysis
- K is dialyzer clearance
- T is dialysis time
- V is volume distribution of urea

References

1. Mathew AT, Fishbane S, Obi Y, Kalantar-Zadeh K. Preservation of residual kidney function in hemodialysis patients: reviving an old concept. Kidney Int. 2016;90 (2):262-271.

2. Mathew AT, Obi Y, Rhee CM, Chou JA, Kalantar-Zadeh K. Incremental dialysis for preserving residual kidney function-Does one size fit all when initiating dialysis? Semin Dial. 2018;31 (4):343-352.

3. Ghahremani-Ghajar M, Rojas-Bautista V, Lau WL, et al. Incremental Hemodialysis: The University of California Irvine Experience. Semin Dial. 2017;30 (3):262-269.

4. Garofalo C, Borrelli S, De Stefano T, et al. Incremental dialysis in ESRD: systematic review and meta-analysis. J Nephrol. 2019

5. Shah AP, Kalantar-Zadeh K, Kopple JD. Is there a role for ketoacid supplements in the management of CKD? Am J Kidney Dis. 2015;65 (5):659-673.

6. Jiang N, Qian J, Sun W, et al. Better preservation of residual renal function in peritoneal dialysis patients treated with a low-protein diet supplemented with keto acids: a prospective, randomized trial. Nephrol Dial Transplant. 2009;24 (8):2551-2558.

7. Whitehead AL, Julious SA, Cooper CL, Campbell MJ. Estimating the sample size for a pilot randomised trial to minimise the overall trial sample size for the external pilot and main trial for a continuous outcome variable. Stat Methods Med Res. 2016 Jun;25(3):1057-73.

8. Bolasco P, Cupisti A, Locatelli F, Caria S, Kalantar-Zadeh K. Dietary Management of Incremental Transition to Dialysis Therapy: Once-Weekly Hemodialysis Combined With Low-Protein Diet. J Ren Nutr. 2016;26 (6):352-359.

9. Obi Y, Streja E, Rhee CM, et al. Incremental Hemodialysis, Residual Kidney Function, and Mortality Risk in Incident Dialysis Patients: A Cohort Study. Am J Kidney Dis. 2016;68 (2):256-265.

10. KDOQI Clinical Practice Guideline for Hemodialysis Adequacy: 2015 update. Am J Kidney Dis. 2015;66 (5):884-930.
